# Phosphatidylcholine Supply to Peroxisomes of the Yeast *Saccharomyces cerevisiae*

**DOI:** 10.1371/journal.pone.0135084

**Published:** 2015-08-04

**Authors:** Vid V. Flis, Ariane Fankl, Claudia Ramprecht, Günther Zellnig, Erich Leitner, Albin Hermetter, Günther Daum

**Affiliations:** 1 Institute of Biochemistry, Graz University of Technology, NAWI Graz, Graz, Austria; 2 Institute of Plant Sciences, University of Graz, NAWI Graz, Graz, Austria; 3 Institute of Analytical Chemistry and Food Technology, Graz University of Technology, NAWI Graz, Graz, Austria; INRA, FRANCE

## Abstract

In the yeast *Saccharomyces cerevisiae*, phosphatidylcholine (PC), the major phospholipid (PL) of all organelle membranes, is synthesized via two different pathways. Methylation of phosphatidylethanolamine (PE) catalyzed by the methyl transferases Cho2p/Pem1p and Opi3p/Pem2p as well as incorporation of choline through the CDP (cytidine diphosphate)-choline branch of the Kennedy pathway lead to PC formation. To determine the contribution of these two pathways to the supply of PC to peroxisomes (PX), yeast mutants bearing defects in the two pathways were cultivated under peroxisome inducing conditions, i.e. in the presence of oleic acid, and subjected to biochemical and cell biological analyses. Phenotype studies revealed compromised growth of both the *cho20Δopi3Δ* (mutations in the methylation pathway) and the c*ki1Δdpl1Δeki1Δ* (mutations in the CDP-choline pathway) mutant when grown on oleic acid. Analysis of peroxisomes from the two mutant strains showed that both pathways produce PC for the supply to peroxisomes, although the CDP-choline pathway seemed to contribute with higher efficiency than the methylation pathway. Changes in the peroxisomal lipid pattern of mutants caused by defects in the PC biosynthetic pathways resulted in changes of membrane properties as shown by anisotropy measurements with fluorescent probes. In summary, our data define the origin of peroxisomal PC and demonstrate the importance of PC for peroxisome membrane formation and integrity.

## Introduction

In the yeast *S*. *cerevisiae* peroxisomes are important organelles for growth on fatty acids and alkaline media. In yeast and in plant cells, the β-oxidation of fatty acids is localized to peroxisomes, whereas in mammalian cells also mitochondria (M) are capable of performing fatty acid degradation. Peroxisomes are also important due to the fact that they harbor oxidative and detoxifying reactions involving oxygen and hydrogen peroxide as substrates [1].

It is believed that peroxisomes originate from the endoplasmic reticulum [2–4]. How this happens is still a matter of debate. Once the peroxisomes have come to maturation, they can divide autonomously [5]. Little is known about the phospholipid composition and especially the supply of phospholipids to peroxisomes. Similar to other biomembranes, peroxisomal membranes contain four major classes of phospholipids, namely phosphatidylserine (PS), phosphatidylethanolamine (PE), phosphatidylcholine (PC) and phosphatidylinositol (PI) [6–8]. In mitochondria, cardiolipin (CL) is also a major phospholipid component. In almost all yeast membranes, PC and PE are present at 60–70% of total phospholipids [9].

Studies from our lab had shown, that PE in yeast cells grown on oleate media can be supplied to peroxisomes from three different sites of synthesis, namely the mitochondria, the endoplasmic reticulum and the Golgi apparatus [7, 10]. First, PE can be provided by the mitochondrial PS decarboxylase Psd1p; second, the vacuolar Psd2p produces PE for peroxisomes; and finally, the CDP-ethanolamine branch of the so-called Kennedy pathway synthesizes a portion of PE destined for peroxisomes. Ethanolamine required for this pathway can be used from external sources. Alternatively, phosphoethanolamine can be introduced into this pathway through degradation of sphingolipids [11]. PC supply to peroxisomes of the yeast *S*. *cerevisiae* has not yet been studied in detail.

**Fig 1:**
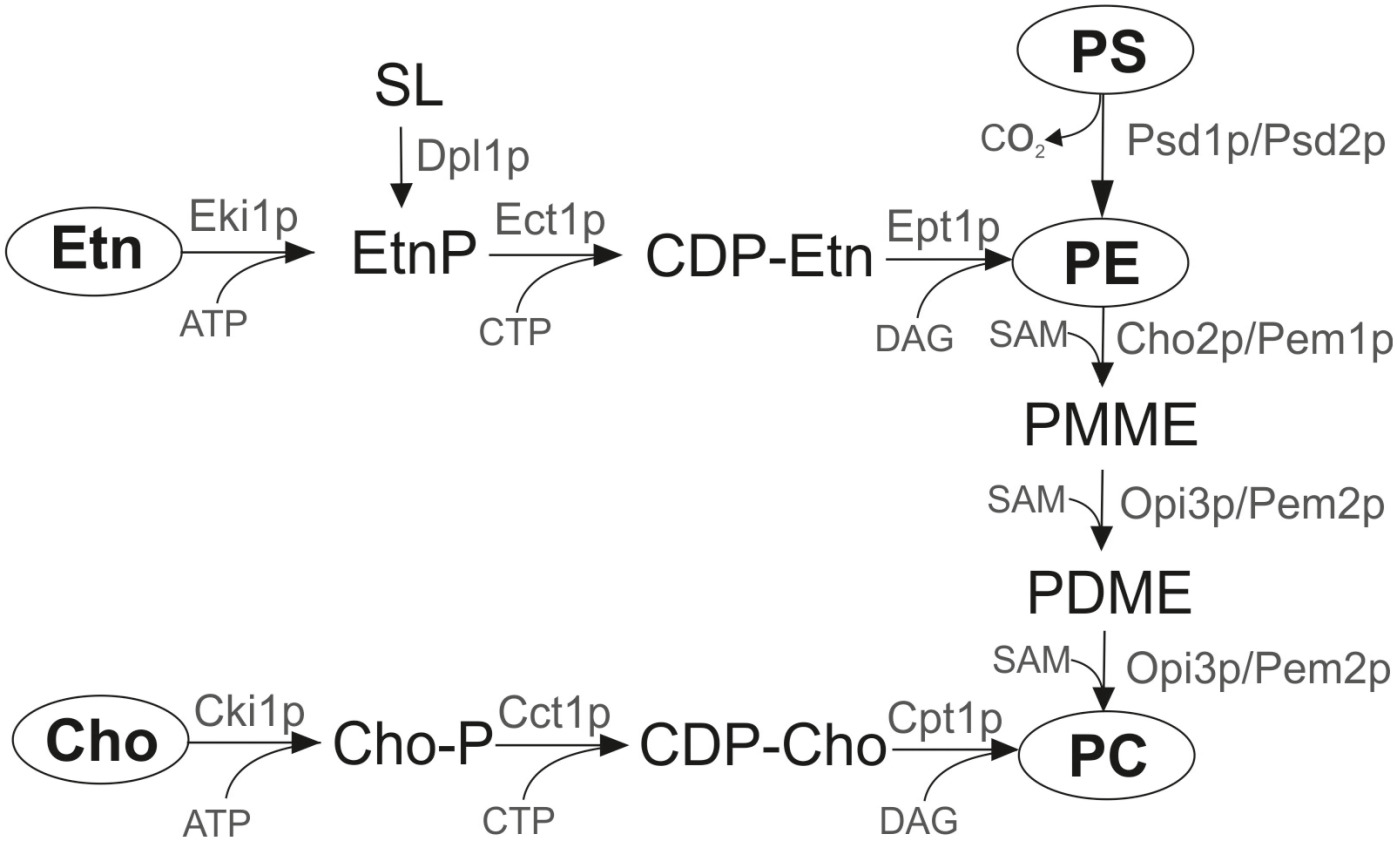
Pathways of phosphatidylcholine biosynthesis in the yeast *S*. *cerevisiae*. Cct1, cholinephosphate cytidylyltransferase; CDP-Cho, cytidine-diphosphocholine, CDP-Etn, cytidine-diphosphoethanolamine; Cho, Choline; Cho2, phosphatidylethanolamine methyltransferase; Cho-P, phosphocholine; Cki1, choline kinase; Cpt1, cholinephosphotransferase; DAG, diacylglycerol; Dpl1, sphingosine phosphate lyase; Ect1, phosphoethanolamine cytidylyltransferase; Eki1, ethanolamine kinase; Ept1, sn-1,2-diacylglycerol ethanolamine- and cholinephosphotranferase; Etn, ethanolamine; Etn-P, phosphoethanolamine; PC, phosphatidylcholine; PE, phosphatidylethanolamine; PDME, phosphatidyldimethylethanolamine; PMME phosphatidylmonomethylethanolamine; PS, phosphatidylserine; Psd1, phosphatidylserine decarboxylase 1; Psd2, phosphatidylserine decarboxylase 2; Opi3, methylene-fatty-acyl-phospholipid synthase; SAM, S-adenosyl-L-methionine; SL, sphingolipids.

In *S*. *cerevisiae* two main pathways of PC production exist: (i) the methylation pathway and (ii) the CDP-choline branch of the Kennedy pathway (Fig 1) [10]. In the methylation pathway, PC is produced from PE which is synthesized either from PS or through the CDP-ethanolamine pathway. Aminoglycerophospholipid synthesis starts with the formation of PS in the endoplasmic reticulum by phosphatidylserine synthase Pss1p/Cho1p [12]. PS can then be decarboxylated by two identical reactions catalyzed by the mitochondrial phosphatidylserine decarboxylase 1 (Psd1p), or by Psd2p located to the vacuole. PE is then methylated through three steps by the methyltransferases Cho2p/Pem1p and Opi3p/Pem2p using S-adenosyl-L-methionine (SAM) as co-substrate [10]. The second pathway of PC production in the yeast is the CDP-choline branch of the Kennedy pathway. In the CDP-choline pathway, externally added or endogenous choline is stepwise incorporated through phosphorylation by choline kinase and activation with CTP by phosphocholine cytidyltransferase. In the last step, phosphocholine is transferred from CDP-choline to diacylglycerol (DAG) by choline phosphotransferase and PC is formed [13].

While PC is the most abundant eukaryotic aminoglycerophospholipid [14] and important for the structure of membranes because of its cylindrical shape [15], little is known about the process of distribution of this lipid within the cell. The specific aim of the present study was to investigate routes of PC supply from its different sites of synthesis to peroxisomes of the yeast *S*. *cerevisiae* grown on oleate media, i.e. under peroxisome inducing conditions. To address this question we initiated studies with increasing specificity and analyzed growth phenotype, lipid composition and membrane properties of mutants compromised in the biosynthesis of PC. These studies allowed us to identify the distribution of PC supply to peroxisomes and other organelles of the two mutants compromised in the specific biosynthetic pathway. The second focus of this study was on the role of PC as a component of peroxisomal membranes from the yeast *S*. *cerevisiae*. We show that the presence of PC in peroxisomal membranes is important for membrane properties. Especially the PC to PE ratio was identified as an important parameter for the biophysical status of the membrane.

## Materials and Methods

### Strains and culture conditions

Yeast strains used in this study are described in Table 1. Cells were cultivated in YPD medium (1% yeast extract, 2% peptone and 2% glucose) to the stationary phase. For induction of peroxisomes, cultures were inoculated to an OD_600_ of 0.1 in YPO media containing 0.3% yeast extract, 0.5% peptone, 0.5% potassium dihydrogen phosphate, pH 6, 0.1% oleic acid (herbal oleic acid pure; Merck, Darmstadt, Germany), 0.2% Tween 80 and 0.1% glucose. Cells were grown to the late logarithmic phase. It has to be noted that YPD and YPO media contain low amounts of ethanolamine and choline. It also has to be noted that oleic acid preparations used routinely as carbon source contained impurities of margaric acid, myristic acid, stearic acid, palmitic acid, palmitoleic acid, linoleic acid and linolenic acid. Growth of the different strains on liquid media was followed by measuring the OD_600_.

**Table 1:** Yeast strains used in this study.

Name	Genotype	Origin
BY4742 (wild type)	MAT*α his3Δ 1; leu2Δ 0; lys2Δ 0; ura3Δ 0*	Euroscarf (Frankfurt, Germany)
*cki1Δdpl1Δeki1Δ*	MATα *his3Δ200*, *leu2Δ1 trp1Δ63*, *ura3-52*, *cki1Δ*::*HIS3*, *dpl1Δ*::*LEU2*, *eki1Δ*::*TRP1*	[16]
*cho2Δopi3Δ*	*MATα his3Δ1; leu2Δ0; lys2Δ0; ura3Δ0; cho2*::*kanMX4*; *opi3*::*kanMX4*	Kindly provided by K. Athenstaedt
*cho2Δ*	MAT*α his3Δ 1; leu2Δ 0; lys2Δ 0; ura3Δ 0 cho2Δ*::*KanMX4*	Euroscarf (Frankfurt, Germany)
*opi3Δ*	MAT*α his3Δ 1; leu2Δ 0; lys2Δ 0; ura3Δ 0 opi3Δ*::*KanMX4*	Euroscarf (Frankfurt, Germany)

### Isolation of peroxisomes, mitochondria and microsomes

For cell fractionation and isolation of peroxisomes, mitochondria and microsomes (endoplasmic reticulum) late exponential cultures of *S*. *cerevisiae* grown on YPO media were used. Cells were harvested and spheroplasted as described by Daum et al. [17]. 2 mg Zymolyase 20T were used per 1 g wet cell weight. Cells were homogenized on ice with a Dounce homogenizer in breaking buffer (5 mM MES; 1 mM KCl; 0.6 M sorbitol and 0.5 mM EDTA, pH 6.0-KOH) with 1 mM phenylmethanesulfonylfluoride (PMSF) added as protease inhibitor. Cell debris and nuclei were removed by centrifugation at 5000 rpm for 5 min. The resulting pellet was collected and subjected to two further rounds of resuspension in breaking buffer, homogenizing and centrifugation. The combined supernatants were centrifuged at 13,000 rpm in an SS34 rotor (Sorvall) for 30 min.

The pellets containing peroxisomes and mitochondria were collected and gently resuspended in a small Dounce homogenizer in breaking buffer plus 1 mM PMSF and centrifuged at low speed (3000 rpm) for 5 min to remove residual cellular debris. The supernatant containing the microsomal fraction was centrifuged at 15,000 rpm for 10 min. The pellet was resuspended in breaking buffer and loaded for further purification on a Nycodenz gradient (17%- 24%- 35%; w/v) in 5 mM MES-KOH, pH 6.0, 1 mM KCl, and 0.24 M sucrose. Centrifugation was carried out in a swing out rotor (Sorvall AH-629) at 26,000 rpm for 90 min. A colorless-white band containing peroxisomes was collected with a syringe at the bottom, diluted in 4 volumes of breaking buffer and sedimented for 15 min at 15,000 rpm in an SS34 rotor. The procedure was repeated for mitochondria which formed a separate band at the top of the tube in the density gradient. The supernatant of the previous step containing the microsomal fraction was also centrifuged with an SS34 rotor for 45 min at 18,000 rpm to obtain a fraction containing the endoplasmic reticulum. The pellet containing the endoplasmic reticulum was collected. All organelles were stored at -70°C for further analysis.

### Protein analysis

Proteins from isolated subcellular fractions were precipitated with trichloroacetic acid (TCA) at a final concentration of 10% for 1 h at 4°C. For protein quantification, the pellet was solubilized in 0.1% SDS, 0.1 M NaOH and analyzed by the method of Lowry et al. [18] using bovine serum albumin as a standard. Proteins were separated by SDS-PAGE (sodium dodecyl sulfate polyacrylamide gel electrophoresis) as described [19]. Electrophoresis was performed with 12.5% separation gels, and SDS-PAGE was carried out at 24 mA for 1.5 h. For studies of protein localization, Western blot analysis was performed [20] with primary rabbit antibodies directed against yeast Fox1p, Por1p, Cytb2p and ER-40kDa protein. Immunoreactive proteins were visualized by ELISA using a peroxidase-linked secondary antibody (Sigma) following the manufacturer’s instructions (SuperSignal, Pierce Chemical Company, Rockford, IL, USA).

### Lipid analysis

Total phospholipids were extracted from homogenate (H), endoplasmic reticulum, mitochondria and peroxisomes by the method of Folch et al. [21]. Total phospholipids were separated from non-polar lipids by one-dimensional thin layer chromatography (TLC) on silica gel plates (Merck, Darmstadt, Germany) using light petroleum/diethyl ether/acetic acid (35:15:1, per vol.) as solvent. Lipid bands were stained with iodine vapor, scrapped off the plate and quantified by the method of Broekhuyse [22]. For total phospholipid analysis 0.8 to 1 mg protein was used.

Individual phospholipids were separated by two-dimensional TLC on silica gel plates (Merck, Darmstadt, Germany) using chloroform/methanol/25% NH_3_ (65:35:5, per vol.) as first solvent, and chloroform/acetone/methanol/acetic acid/water (50:20:10:10:5; per vol.) as second solvent. Lipid bands were visualized with iodine vapor, scrapped off the plate and quantified by the method of Broekhuyse [22]. For the quantification of individual phospholipids samples containing 1 mg protein were used.

Fatty acids were analyzed by gas liquid chromatography (GLC). Lipid extracts prepared as described above were incubated with 2.5% sulfuric acid in methanol at 85°C for 90 min [23]. After incubation and cooling of the samples water was added, and fatty acids converted to methyl esters were extracted with light petroleum. Fatty acid methyl esters were separated using a Hewlett-Packard 6890-Gas-Chromatograph equipped with a HP-INNO Wax capillary column (15 m × 0.25 mm i.d. × 0.50 μm film thicknesses) and helium as carrier gas (20 min at 200°C, 10 min to 280°C, 15 min at 300°C). Fatty acids were identified by comparison to commercial fatty acid methyl ester standards (NuCheck, Inc., Elysian, MN, USA).

Free fatty acids were extracted from homogenate, endoplasmic reticulum, mitochondria and peroxisomes by the method of Folch et al. [21]. Oleic acid was separated from other free fatty acids by one-dimensional thin layer chromatography (TLC) on silica gel plates (Merck, Darmstadt, Germany) using light petroleum/diethyl ether/acetic acid (35:15:1, per vol.) as first solvent and light petroleum/diethyl ether (49:1) as second solvent. Free fatty acid bands were visualized by dipping TLC plates into a charring solution (0.63 g MnCl_2_ x 4 H_2_O, 60 ml H_2_O, 60 ml MeOH and 4 ml concentrated H_2_SO_4_) and incubating for 30 min at 100°C. Bands were quantified by scanning at 400 nm using a CAMAG TLC Scanner 3.

Sterols from whole cells or subcellular fractions were identified and quantified by gas liquid chromatography/mass spectrometry (GLC—MS) [24, 25]. In brief, a mixture of 0.6 ml methanol (Merck), 0.4 ml 0.5% (w/v) pyrogallol (Fluka) dissolved in methanol and 0.4 ml 60% (w/v) aqueous KOH solution was placed into 15 ml Pyrex tubes. As an internal standard 5 μl of cholesterol stock solution (2 mg/ml) were added. For analyzing sterols, organelles containing 0.5 to 1.0 mg protein were used. The respective amount of organelles was added to the reaction mixture, and tubes were heated in a sand bath for 2 h at 90°C. Then, lipids were extracted three times with 1 ml n-heptane, each. The upper phase was transferred into a new tube and the lower phase was re-extracted. The combined upper phases were dried under a stream of nitrogen, and lipids were dissolved in 10 μl pyridine. After adding 10 μl N´O´-bis(trimethylsilyl)-trifluoracetamide (Sigma) samples were diluted with 50 μl ethylacetate and analyzed by GLC-MS. GLC—MS was performed on an HP 5890 Gas-Chromatograph equipped with a mass selective detector HP 5972, using an HP5-MS capillary column (30 m × 0.25 mm, 0.25 μm film thickness). Aliquots of 1 μl were injected in the splitless mode at 270°C injection temperature with helium as carrier gas at a flow rate of 0.9 ml/min in constant flow mode. The following temperature program was used: 1 min at 100°C, 10°C/min to 250°C, and 3°C/min to 310°C. Mass spectra were acquired in the scan mode (scan range 200–550 amu) with 3.27 scans per second. Sterols were identified based on their mass fragmentation pattern.

For non-polar lipid analysis, lipids from yeast cells were extracted as described above. Lipids were applied to Silica Gel 60 plates, and chromatograms were developed in an ascending manner by a two-step developing system. First, chromatograms were developed using light petroleum/diethyl ether/acetic acid (70:30:2; per vol.) to two thirds of the plate. After drying, plates were further developed to the top using light petroleum/diethyl ether (49:1; v/v) as the second solvent system. Chromatograms for DAG analysis were developed using chloroform/acetone/ acetic acid (45:4:0.5; per vol.). To visualize separated bands, TLC plates were dipped into a charring solution consisting of 0.63 g MnCl_2_ × 4H_2_O, 60 ml water, 60 ml methanol and 4 ml concentrated sulfuric acid, briefly dried and heated at 100°C for 20 min. Then, lipids were quantified by densitometric scanning at 400–650 nm with diolein and triolein as standards using a Shimadzu dual-wave length chromatoscanner CS-930.

### Fluorescence anisotropy measurements

Isolated organelles (100 μg) were suspended in breaking buffer, pH 6 (5 mM MES, 1 mM KCl 0.5 mM EDTA, 0.6 M sorbitol). After addition of an organic solution of diphenylhexatriene (DPH) at a molar ratio of 1:50 (probe to phospholipid), mixtures were incubated for 5 min at 30°C. Samples were kept in the dark until fluorescence measurements were carried out using a Shimadzu RF 540 spectrofluorimeter equipped with polarizers in the excitation and emission light path. Excitation and emission wavelengths for DPH were 350 nm and 452 nm, respectively (slit width 10 nm). Fluorescence intensities were corrected for background fluorescence and light scattering from the unlabeled sample. The fluorescence anisotropy was calculated according to the equation r = (I_║_-I_┴_) / (I_║_+2*I_┴_). The values of I_║_ and I_┴_ are measured emission intensities parallel and perpendicular to the vertical polarization plane of the excitation light [26].

### Electron microscopy

For electron microscopic examination, cell precultures were grown under aerobic conditions at 30°C on YPD medium containing 2% glucose as carbon source. Cells were diluted to an OD_600_ of 0.1 in fresh YPO medium and grown to the late exponential phase. Then, cells were harvested by centrifugation and washed twice with 0.5% BSA (fatty acid free) and 3 times with H_2_O. Washed cells were fixed for 5 min in a 1% aqueous solution of KMnO_4_ at room temperature, washed with double distilled water and fixed again in a 1% aqueous solution of KMnO_4_ for 20 min. Fixed cells were washed three times in distilled water and incubated in 0.5% aqueous uranyl acetate for the first three hours with shaking at room temperature and afterwards overnight at 4°C. Samples were then dehydrated for 20 min in a graded series of 50%, 70%, 90% and 100% ethanol, each. Pure ethanol was then changed to propylene oxide, and specimen were gradually infiltrated with increasing concentrations of Agar 100 epoxy resin (30%, 50%, 70% and 100%) mixed with propylene oxide for a minimum of 3 h per step. Samples were embedded in pure, fresh Agar 100 epoxy resin and polymerized at 60°C for 48 h. Ultra-thin sections of 80 nm were stained with lead citrate and viewed with a Philips CM 10 transmission electron microscope.

## Results

### Growth characteristics of yeast mutants compromised in one of the two phosphatidylcholine biosynthetic pathways

**Fig 2:**
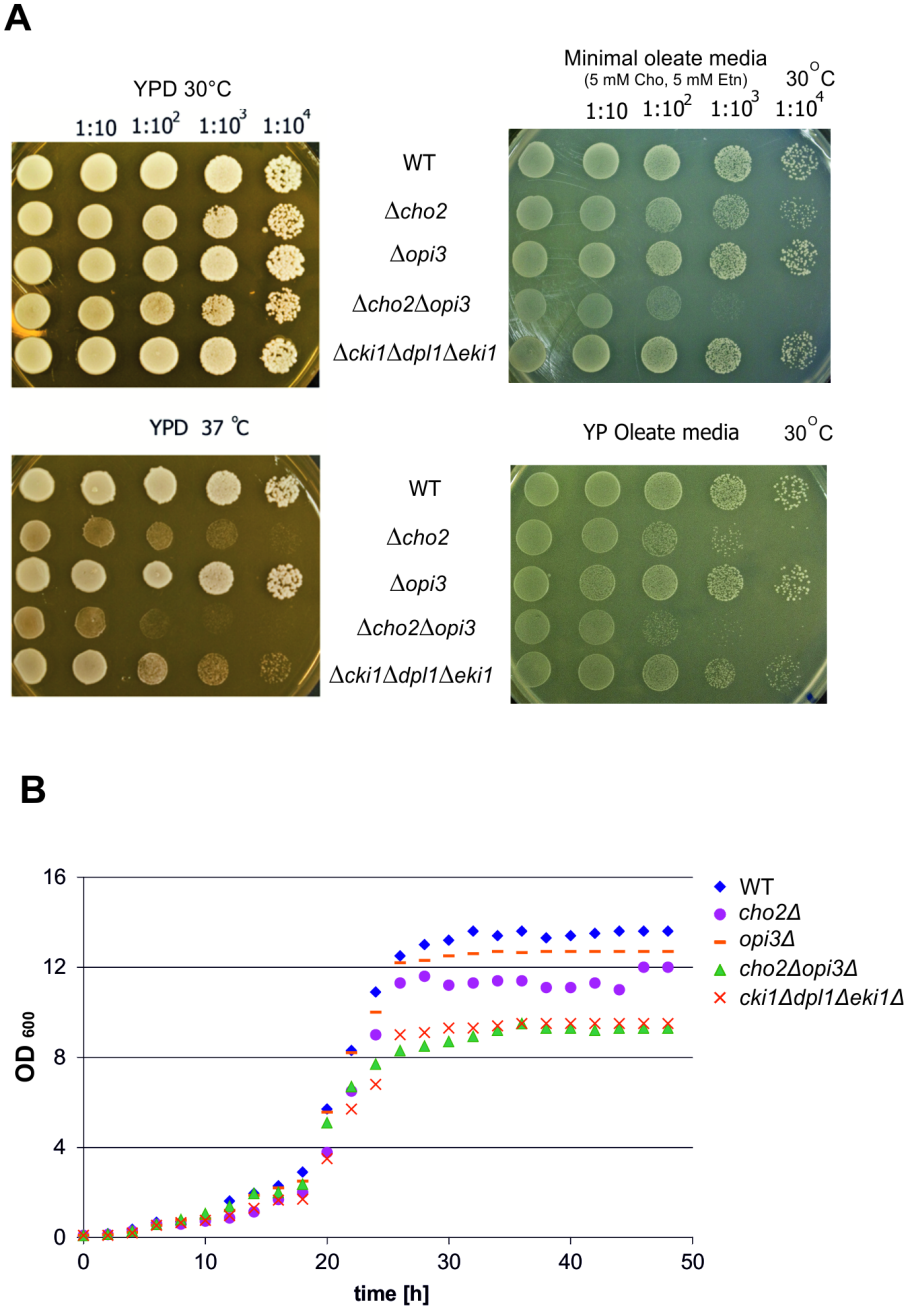
Growth phenotype of wild type, *cho2Δ*, *opi3Δ*, *cho2Δopi3Δ* and *cki1Δdpl1Δeki1Δ* yeast strains. (A): Drop test on YPD plates (30°C and 37°C); on minimal oleate media containing choline and ethanolamine; and on YPO plates are shown. (B) Growth of liquid cultures on YPO.

To analyze the influence of mutations in PC synthesis on growth of yeast cells on different carbon sources drop tests on YPD and YPO agar plates (Fig 2A) were performed and cells were cultivated in liquid media (Fig 2B). These tests showed that strains bearing defects in the CDP-choline pathway of PC synthesis grew normally on YPD at 30°C. The *cho2*Δ*opi3*Δ mutant showed only slight growth defects on YPD at 30°C. At a temperature of 37°C on YPD, however, *cho2*Δ, *cho2*Δ*opi3*Δ and the *cki1*Δ*dpl1*Δ*eki1*Δ mutants exhibited a growth defect. As shown in Fig 2A growth of the *cho2*Δ*opi3*Δ mutant was more affected than growth of *cki1*Δ*dpl1*Δ*eki1*Δ. On minimal media containing oleate as carbon source and supplemented with 5 mM choline and 5 mM ethanolamine the *cho2*Δ*opi3*Δ mutant grew markedly worse than wild type (see Fig 2A). Under these conditions only a slight growth defect of *cho2*Δ was observed, and growth of the *cki1*Δ*dpl1*Δ*eki1*Δ mutant was practically not affected. Surprisingly, on YPO rich media the growth defect of *cho2*Δ, *cho2*Δ*opi3*Δ and the *cki1*Δ*dpl1*Δ*eki1*Δ mutants was more pronounced than on minimal oleate containing media. This effect may be due to the limiting amount of ethanolamine and choline present in YPO. At 37°C, mutant strains did not grow at all on minimal oleate media supplemented with choline and ethanolamine, although the wild type strain showed normal growth (data not shown).

The mutant strains *cho2*Δ*opi3*Δ and *cki1*Δ*dpl1*Δ*eki1*Δ showed similar growth in liquid media (Fig 2B), but clear growth defects compared to the wild type strain. The *cho2*Δ mutant was only slightly affected, and the *opi3*Δ mutant behaved almost like wild type.

Taken together, these data suggest that under the chosen growth conditions defects in PE methylation result in more severe growth defects than mutations in the CDP-choline pathway of PC formation on solid media. Noteworthy, these defects become more evident under conditions of elevated temperature and on oleate as carbon source than on YPD under standard conditions. In liquid media the two mutant strains had similar growth defects.

### Electron microscopy of yeast cells bearing defects in phosphatidylcholine biosynthesis

**Fig 3:**
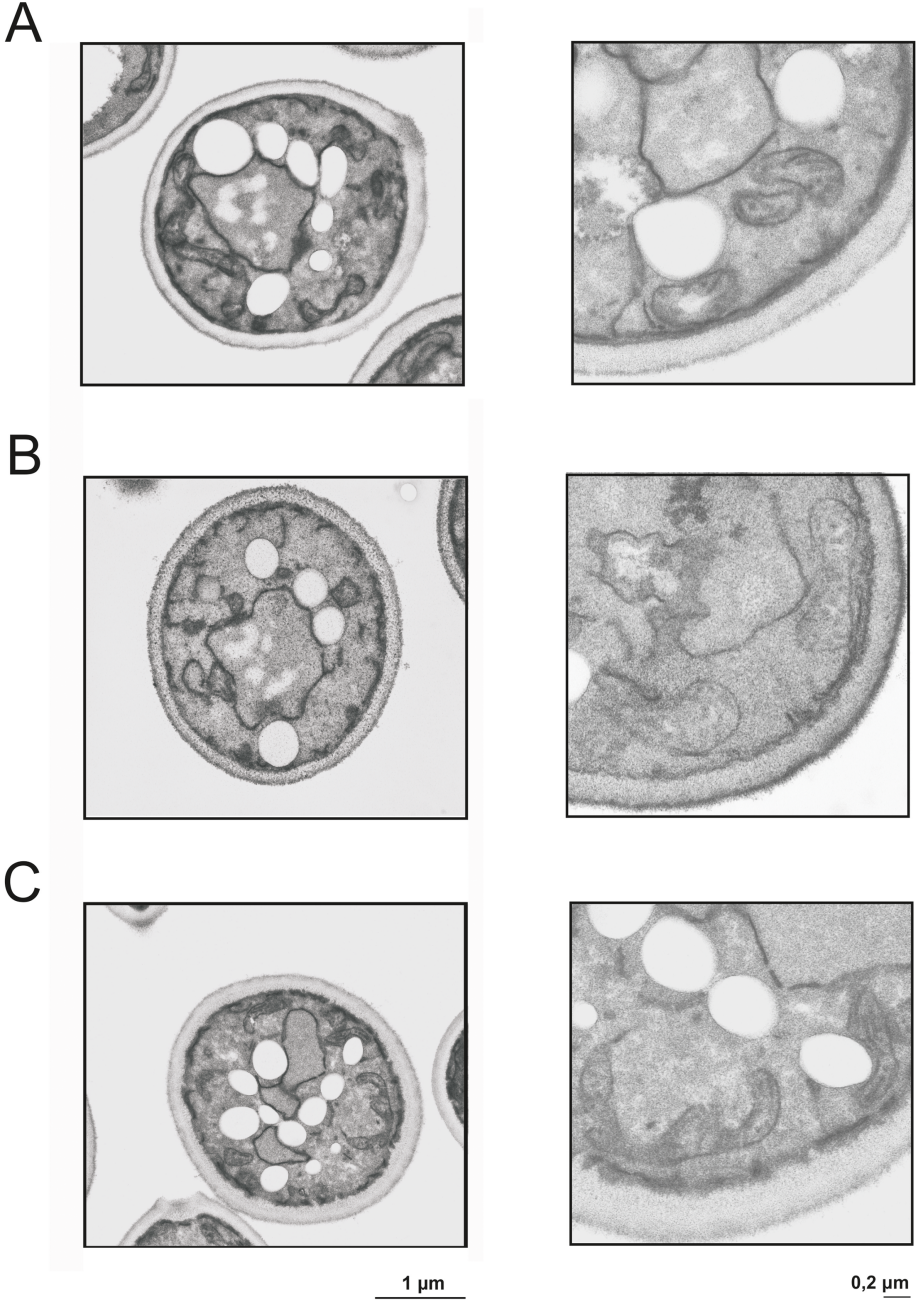
Transmission electron microscopy of wild type and mutant strains. Ultrathin sections of chemically fixed *S*. *cerevisiae* yeast cells are shown. For experimental details see Materials and Methods section. Mitochondria are marked with arrows. Wild type (A), *cki1Δdpl1Δeki1Δ* (B), and *cho2Δopi3Δ* (C) were grown on YPO to induce formation of peroxisomes.

To investigate possible morphological changes caused by mutations in the different PC biosynthetic pathways wild type, *cho2*Δ*opi3*Δ and the *cki1*Δ*dpl1*Δ*eki1*Δ mutant strains grown on oleate media were subjected to electron microscopic inspection (Fig 3). Growth of yeast cells on fatty acids as carbon source not only induces peroxisome proliferation, but also causes enhanced formation of lipid droplets. These droplets mainly consist of non-polar lipids and are a storage compartment for triacylglycerols and steryl esters [27]. Data from our group [7] and from other laboratories had indicated that peroxisomes have a tendency to associate with other subcellular compartments, especially the endoplasmic reticulum [28, 29], mitochondria [30–32] and lipid droplets [33]. Especially the contact between peroxisomes and lipid droplets was considered to be relevant for the supply of fatty acids as a substrate for β-oxidation to peroxisomes, although biochemical evidence for such a route is missing. The association of peroxisomes with mitochondria and the endoplasmic reticulum [29] may be more important for the biogenesis of peroxisomes including supply of lipids from the respective sites of synthesis. Electron micrographs (see Fig 3), however, did not show fundamental changes of the cellular structure caused by mutations in the PC biosynthetic pathways. One obvious difference between the strains analyzed was the decreased number of lipid droplets in the *cki1Δdpl1Δeki1Δ* mutant. These data are in line with previous results from our laboratory [34] showing that a *cki1Δdpl1Δeki1Δ* strain grown on YPD medium with glucose as a carbon source had a markedly lower level of triacylglycerols than wild type. The morphology and the size of mitochondria were not much affected by the mutations. Despite the fact that the *cki1Δdpl1Δeki1Δ* mutant contains considerably less phospholipid than wild type (see below) only minor difference in size and area of mitochondria were found (Table 2).

**Table 2:** Structural parameters of mitochondria from different mutant strains. Electron micrographs of the tested strains were obtained as described in the Materials and Methods section. For the analysis of mitochondrial size, circumference and area 70 to 100 mitochondria were tested.

	Size (μm)	Circumference (μm)	Area (μm²)
Wild type	0.50	1.34	0,084
*cki1Δdpl1Δeki1Δ*	0.50	1.41	0,105
*cho2Δopi3Δ*	0.56	1.51	0,087

### Isolation and characterization of peroxisomes

**Fig 4:**
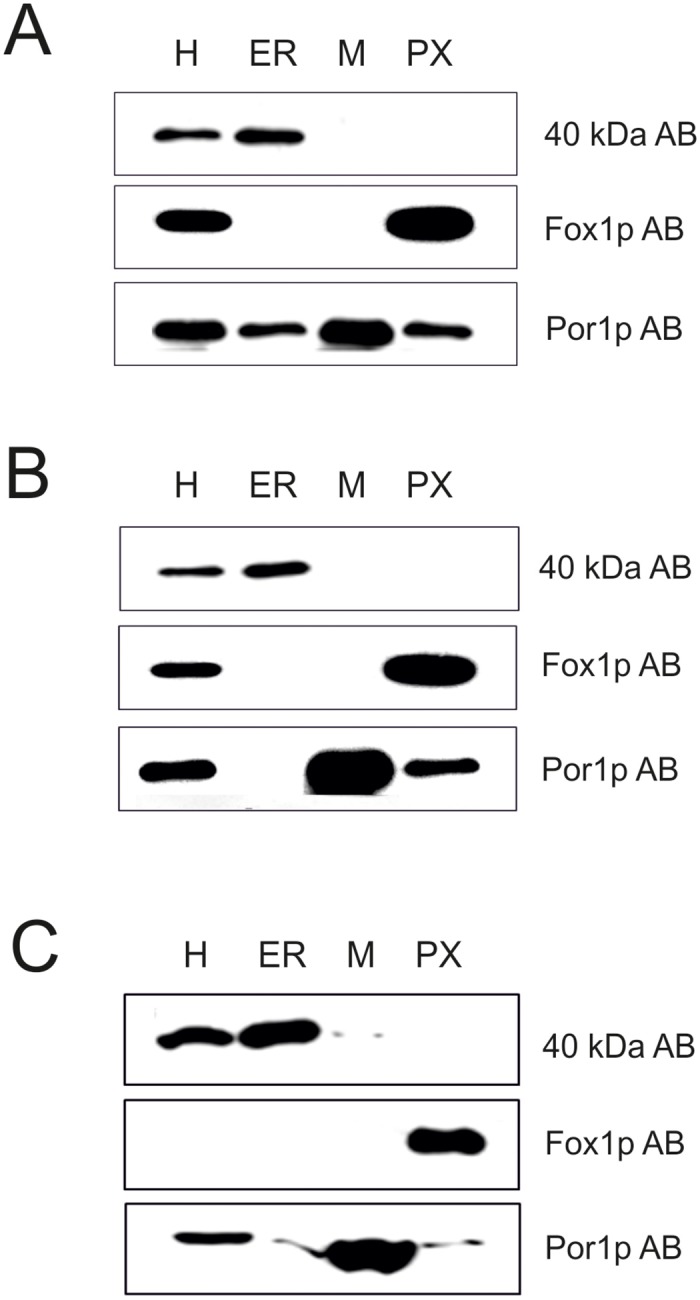
Western blot analysis of subcellular fractions from *S*. *cerevisiae*. Wild type (A) and mutant strains *cki1*Δ*dpl1*Δ*eki1*Δ (B) and *cho2*Δ*opi3*Δ (C) were grown on oleic acid as described in the Materials and Methods section. H (homogenate), ER (endoplasmic reticulum), M (mitochondria) and PX (peroxisomes) were isolated according to standard procedures [35]. For electrophoresis 10 μg protein were loaded onto each lane of the gel.

**Fig 5:**
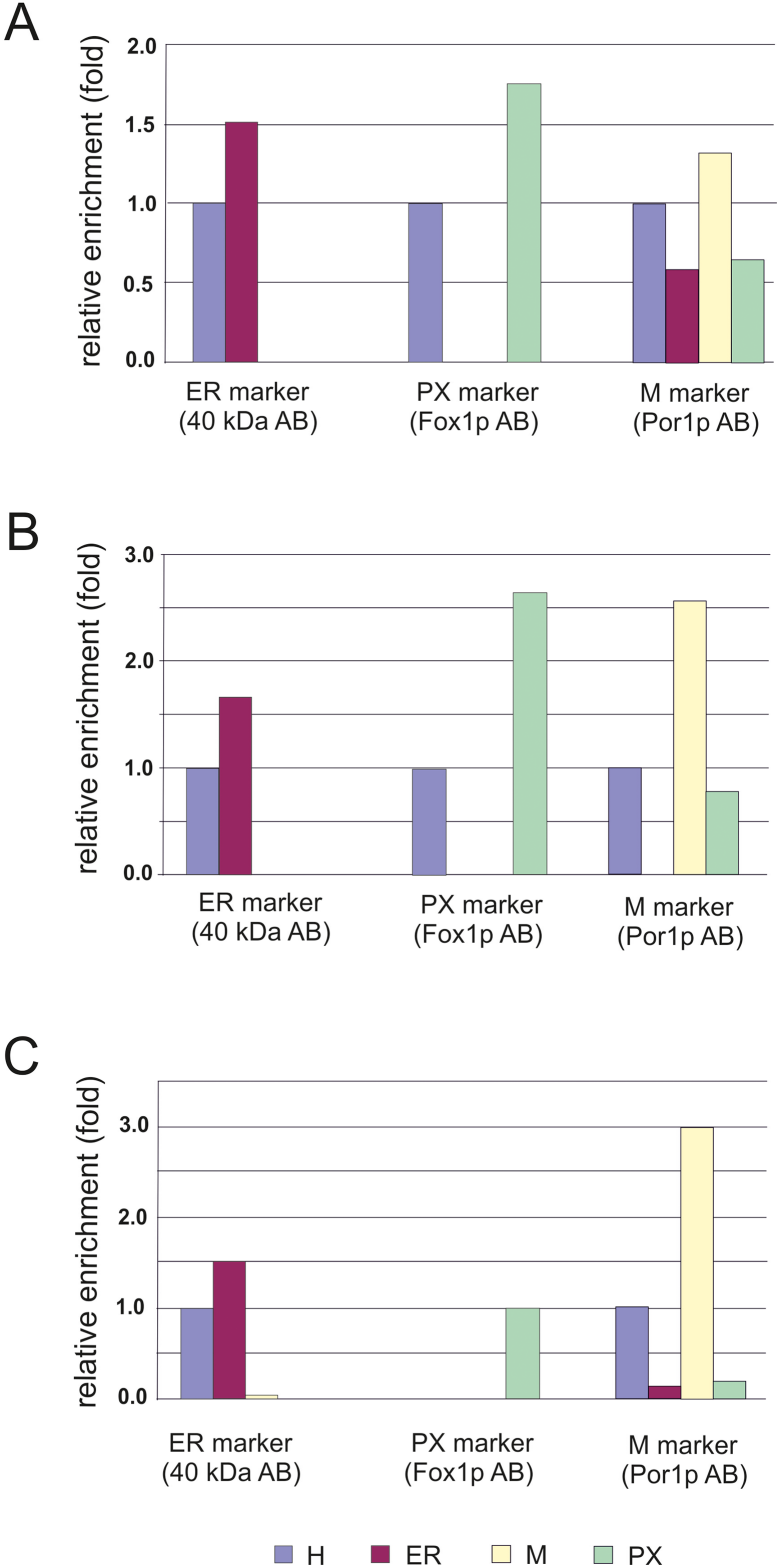
Quantification of Western blot analysis of subcellular fractions from *S*. *cerevisiae*. ER (endoplasmic reticulum), M (mitochondria) and PX (peroxisomes) were compared to the H (homogenate) of the wild type strain (A), the *cki1*Δ*dpl1*Δ*eki1*Δ (B) and the *cho2*Δ*opi3*Δ (C) mutant strains.

Prerequisite for the analysis of effects on the formation of peroxisomes caused by mutations in the two PC biosynthetic pathways was the isolation of these organelles as described previously by Zinser and Daum [35]. The quality of peroxisome preparations was tested by Western blot analysis to determine the enrichment of peroxisomes and cross contamination with other organelles (Fig 4). Antibodies used for Western blot analysis were directed against a 40 kDa protein (endoplasmic reticulum); Fox1p (multifunctional β-oxidation protein from peroxisomal membranes) and Por1p (outer mitochondrial membrane). As can be seen from Fig 4, Fox1p was highly enriched in peroxisomal fractions from wild type cells. The degree of cross-contamination of peroxisomes with other subcellular fractions was low. Impurities caused by co-isolation of mitochondria were small as can be seen from the patterns of Por1p. Western blot analysis was also routinely performed with subcellular fractions from the mutant strains bearing defects in PC synthesis. Cross contaminations were quantified in Fig 5. Contaminations with mitochondria were found in all fractions most likely due to close contact of mitochondria with other organelles. Endoplasmic reticulum and mitochondria were not contaminated by peroxisomes, and no contamination with endoplasmic reticulum (ER) was found with the other isolated organelles.

### Phospholipid analysis of peroxisomes from cells with phosphatidylcholine biosynthetic defects

To characterize the contributions of the two PC biosynthetic pathways of the yeast to the supply of PC to peroxisomes phospholipid patterns from *cki1*Δ*dpl1*Δ*eki1*Δ and *cho2*Δ*opi3*Δ mutants were compared to wild type. To obtain a broader view of the expected effects, we analyzed phospholipids from peroxisomes, endoplasmic reticulum and mitochondria.

**Fig 6:**
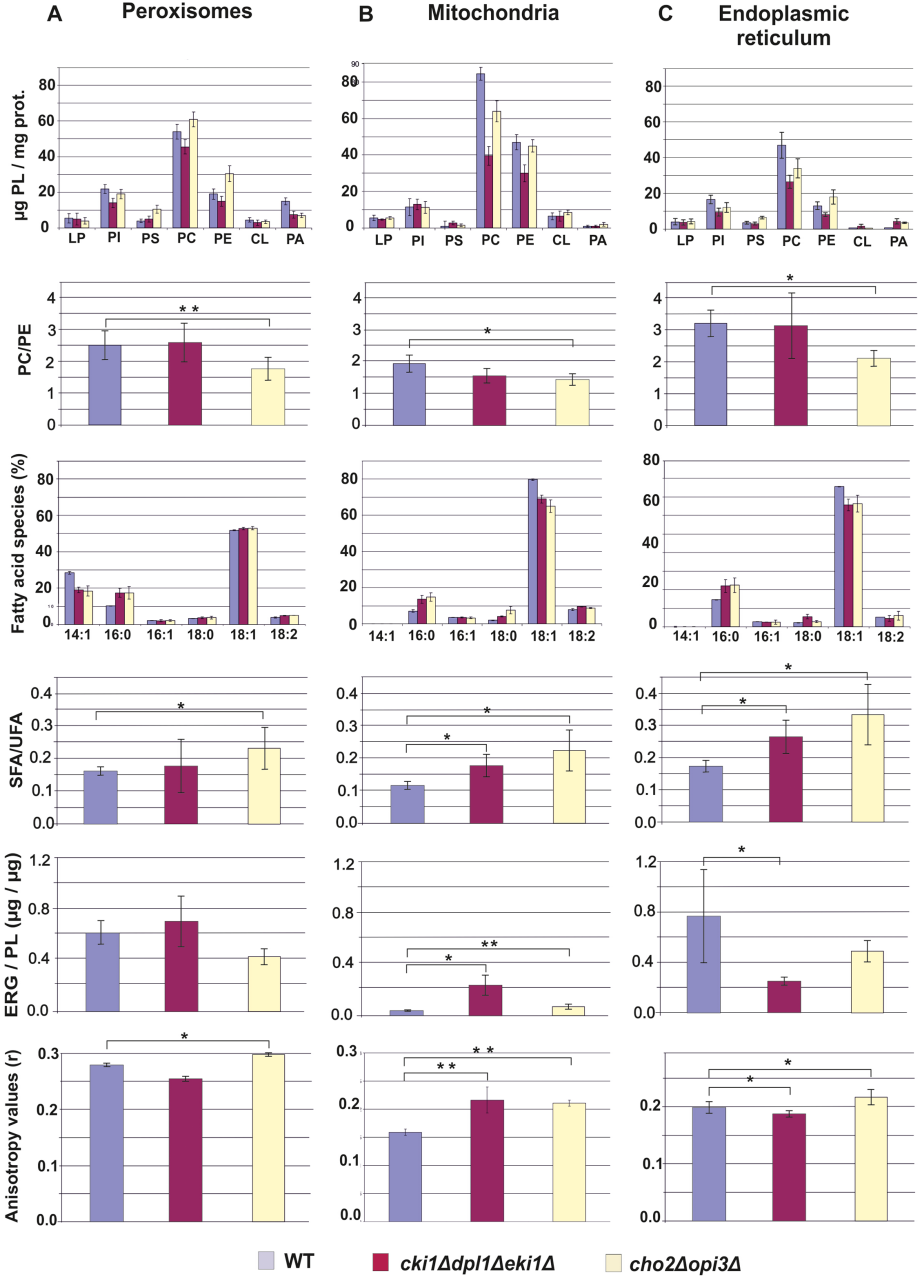
Analysis of organelles from wild type, *cho2Δopi3Δ* and *cki1Δdpl1Δeki1Δ* yeast strains. Yeast strains used were grown on YPO media. Data obtained with isolated peroxisomes (lane A), mitochondria (lane B) and endoplasmic reticulum (lane C) are shown. Line 1: Phospholipid pattern expressed as μg of individual phospholipids per mg protein. LP, lysophospholipids; PI, phosphatidylinositol; PS, phosphatidylserine; PC, phosphatidylcholine; PE, phosphatidylethanolamine; CL, cardiolipin; PA, phosphatidic acid. Line 2: PC to PE ratio in different organelles and strains. Line 3: Fatty acid composition of different organelles and strains. Line 4: Ratio of saturated (SFA) to unsaturated (UFA) fatty acids in different organelles and strains. Line 5: Ergosterol (ERG) to phospholipid (PL) ratio in different organelles and strains. Line 6: Anisotropy values obtained with different organelles and strains. For experimental details see Materials and Methods section. For all experiments two independent biological samples were used which were analyzed 2 to 3 time, each.

A key experiment to understand PC supply to peroxisomes was the analysis of individual phospholipids from wild type (WT), the *cki1Δdpl1Δeki1Δ* mutant and the *cho2Δopi3Δ* mutant (Fig 6). In peroxisomes from the *cho2Δopi3Δ* strain the amount of PC was even slightly increased over wild type, whereas the PC level in peroxisomes from *cki1Δdpl1Δeki1Δ* was slightly decreased. Thus, PC production for peroxisomal membranes through the CDP-choline pathway in the strain lacking enzymes of PE methylation appears to be more efficient than through the methylation pathway. It has to be noted, however, that according to these results both pathways of PC synthesis in the yeast supply PC to peroxisomes, although with slightly different efficiency. Interestingly, however, the PE and PS levels in peroxisomes of the *cho2Δopi3Δ* mutant were markedly higher than in wild type and in the *cki1Δdpl1Δeki1Δ* strain. Some accumulation of PE and PS may have occurred due to the lack of further conversion to PC. In the *cki1Δdpl1Δeki1Δ* strain levels of all phospholipids were found to be lower than in wild type. Results described above led to an interesting effect, namely a decrease in the PC to PE ratio in the *cho2Δopi3Δ* mutant. Taking into account that this ratio may affect membrane properties, the double mutation appears to cause a marked effect. In the *cki1Δdpl1Δeki1Δ* strain the PC to PE ratio was similar to wild type.

Some of the trends described with phospholipids from peroxisomes were also observed with mitochondrial and endoplasmic reticulum membranes, in some cases even more pronounced. Both in mitochondria and in the endoplasmic reticulum *cki1Δdpl1Δeki1Δ* and *cho2Δopi3Δ* mutations caused a decrease of PC levels. Effects with the *cki1Δdpl1Δeki1Δ* triple deletion were more pronounced than with the *cho2Δopi3Δ* double deletion. In mitochondria from both strains, the PC to PE ratio was lower than in wild type, whereas in the endoplasmic reticulum this ratio was more affected by the *cho2Δopi3Δ* double deletion. However, in principle also mitochondria and the endoplasmic reticulum can obtain PC from both sites of synthesis.

Cardiolipin (CL) was also found in peroxisomes, although this lipid has been considered for a long time specific for mitochondria. The CL content measured in peroxisomes occurred mainly from contamination with mitochondrial membranes (71% in wild type; 67% in the *Δcki1Δdpl1Δeki1* mutant. In the *Δcho2Δopi3* mutant only 16% of peroxisomal CL could be attributed to contamination with mitochondria. The remaining amounts of CL have therefore to be considered as “true” peroxisomal.

Mitochondria isolated from the *Δcki1Δdpl1Δeki1* mutant (see Fig 6) contained considerably less phospholipids (PC and PE) than the wild type. As isolated mitochondria were not contaminated with other organelles (see Figs 4 and 5), the purity of samples did not influence the result. Surprisingly, the changes in the phospholipid of strains did not affect the structure of mitochondria (see Fig 3 and Table 2).

### Fatty acid analysis

As phospholipid patterns of the organelle membranes from the three analyzed strains (wild type, PE methylation and CDP-choline mutant) were versatile we also tested changes in the fatty acid patterns of the respective organelles (see Fig 6). Not surprisingly, oleate (C18:1) was the predominant fatty acid in all samples due to the fact that this fatty acid present in the medium is not only used as a carbon source but also as a component for the synthesis of membrane phospholipids. Interestingly, the level of C18:1 in peroxisomes was markedly lower than in the endoplasmic reticulum and mitochondria indicating that some selectivity in the supply of lipids to the different organelle membranes had occurred.

In all three strains tested, the C14:1 fatty acid was only found in peroxisomes but not in mitochondria and in the endoplasmic reticulum. The origin of peroxisomal C14:1 remains unclear. As the oleic acid used as carbon source contained some C14:1, an external origin is very likely. The fact remains, however, that C14:1 accumulated in peroxisomes whereas other organelles were practically devoid of this fatty acid. These findings support some selectivity in the supply of certain lipid species to peroxisomes. C16:0 was present in all three strains and organelles at a level of ~ 10–20%, whereas C16:1 and C18:0 fatty acids were detected only at low concentrations. C18:2 was present in all samples due to impurities of oleic acid samples used as carbon source. The used oleic acid contains max. 5% of C14:1 (myristic acid), 16% of C16:0 (palmitic acid), 8% of C16:1 (palmitoleic acid), 6% of C18:0 (stearic acid), 65–88% of C18:1 (oleic acid) and 18% of C18:2 (linoleic acid).

Although variations in individual fatty acids from the different samples appear to be minor they had a major impact on an important parameter of membrane properties, which is the ratio of saturated to unsaturated fatty acids (see Fig 6). In all organelle samples from *cki1Δdpl1Δeki1Δ* and *cho2Δopi3Δ* this ratio was markedly increased over the wild type. The highest ratio of saturated to unsaturated fatty acids was found in organelles from the *cho2Δopi3Δ* strain.

### Sterol analysis of organelles from cells with phosphatidylcholine biosynthetic defects

In addition to the phospholipid and fatty acid composition, sterols may have an influence on the properties of membranes. For this reason, we compared sterol levels in peroxisomes, mitochondria and endoplasmic reticulum from wild type, *cki1Δdpl1Δeki1Δ* and *cho2Δopi3Δ* (see Fig 6). In all fractions tested the major sterol was ergosterol. In peroxisomes of wild type, 57 μg ergosterol/mg protein was detected. In the *cki1Δdpl1Δeki1Δ* mutant the sterol amount was slightly increased (61 μg ergosterol/mg protein), whereas in the *cho2Δopi3Δ* mutant it was slightly decreased (48 μg ergosterol/mg protein). The more crucial value for membrane properties is the sterol to phospholipid ratio. As can be seen from Fig 6 this value was increased in peroxisomes from *cki1Δdpl1Δeki1Δ* over wild type, whereas it was decreased in *cho2Δopi3Δ*. Compared to peroxisomes, the ergosterol to phospholipid ratio in mitochondria was low. Also in this case the *cki1Δdpl1Δeki1Δ* triple deletion led to a marked increase of this value. In the endoplasmic reticulum the ergosterol to phospholipid ratio was lower in *cki1Δdpl1Δeki1Δ* and *cho2Δopi3Δ* than in wild type.

### Organelle membrane fluidity affected by defects in phosphatidylcholine biosynthetic pathways

Evaluation of anisotropy values obtained with biological membranes is a serious task because many membrane parameters such as the phospholipid pattern, the phospholipid to protein ratio, the PC to PE ratio, the fatty acid pattern, the ratio of saturated to unsaturated fatty acids and the ergosterol to phospholipid ratio may contribute to the overall fluidity/rigidity of a membrane. High ratios of saturated to unsaturated fatty acids, high amounts of ergosterol, low phospholipid to protein ratios and low PC to PE ratios in membranes were considered as parameters that may lead to high anisotropy values indicating high rigidity of membranes. The overall fluidity/rigidity of a membrane is result of a combination of all parameters mentioned.

PC is the classical bilayer forming phospholipid, whereas the non-bilayer forming phospholipid PE may induce curvature of the membrane and disturb the order of the bilayer. Therefore, organelles from strains with compromised PC biosynthesis were good candidates for changed membrane properties. To test organelle membrane rigidity/fluidity, anisotropy measurements using the fluorophore diphenylhexatriene (DPH) were performed. High anisotropy values are indicative of membrane rigidity, whereas low anisotropy is typical for more fluid membranes.

Anisotropy measurements of peroxisomal membranes, endoplasmic reticulum and mitochondria from wild type, *cki1*Δ*dpl1*Δ*eki1*Δ and *cho2*Δ*opi3*Δ mutants are shown in Fig 6. As can be seen, the fluidity of peroxisomal and endoplasmic reticulum membranes of the two mutant strains showed slight differences compared to the membranes of the wild type. In both organelles, the *cki1Δdpl1Δeki1Δ* triple deletion caused a slight decrease of the anisotropy, whereas a slight increase of anisotropy values was observed with *cho2*Δ*opi3*Δ. In general, anisotropy values were lower in the endoplasmic reticulum fraction indicating higher fluidity of these membranes. In mitochondrial membranes, defects in both PC biosynthetic pathways led to higher anisotropy compared to wild type. In this case, differences were more pronounced than in peroxisomes and endoplasmic reticulum indicating that changes in the mitochondrial membrane composition led to a marked increase of membrane rigidity.

The result of the anisotropy measurements of the peroxisomes was surprising, because the ratio of saturated to unsaturated fatty acids was higher in both mutants than in wild type, and the sterol content varied. However, anisotropy (fluidity/rigidity) seemed to correlate with the observed PC to PE ratio in peroxisomal membranes under the mentioned conditions, because low PC to PE ratios led to high anisotropy values, whereas high PC to PE ratios led to low anisotropy values (see Fig 6). The other lipid components (fatty acids and ergosterol) seemed to have less influence on the fluidity/ rigidity of the membrane in this case.

Although we have to be very cautious with the interpretation of these data, two aspects have to be mentioned. First, the amount of sterols in the membranes which were examined was rather low and may not contribute much to the overall rigidity of the membranes. Secondly, the ratio of saturated to unsaturated fatty acids is very low, because more than 85% of fatty acids are unsaturated due to the massive incorporation of oleate into lipids. Thus, small changes in the pattern result in relatively high changes of the ratio. Finally, we are left with a possible effect of changed amounts of PC and PE in peroxisomal membranes, which might result in the observed changes of fluidity/rigidity.

The same arguments as for peroxisomes hold for mitochondria and the endoplasmic reticulum. Also in these samples the effect of fatty acids and sterols may not be crucial for the fluidity of the membranes. Again, the PC to PE ratio can be regarded as a parameter which may influence this membrane property.

## Discussion

Peroxisomes belong to the group of organelles which cannot synthesize all of their own lipids. In mammalian cells, peroxisomes contribute to phospholipid biosynthesis by the formation of ether lipids and to cholesterol biosynthesis (for recent reviews see refs [36] and [37]), but yeast peroxisomes are completely devoid of lipid biosynthetic activities. As a consequence of these facts, peroxisomes rely to a large extend or completely on the supply of lipids from other organelles.

The supply of PE to peroxisomes of the yeast *S*. *cerevisiae* from the three sites of PE synthesis in the mitochondria, the Golgi/vacuole and the endoplasmic reticulum was studied previously in our lab [7]. These investigations revealed that all three pathways of PE synthesis can produce PE for peroxisomes, although with different efficiency. The supply of PC to peroxisomes and the correlation between PC and PE synthesis in the yeast *S*. *cerevisiae* are presented in this study. This process has not been studied before neither with the yeast nor with other cellular systems. As one important result presented here we show that both the methylation pathway and the CDP-choline branch of the Kennedy pathway produce PC destined to peroxisomes, but also to mitochondria and the endoplasmic reticulum. Interestingly, the two pathways exhibit different efficiency in PC formation for peroxisomes, at least under growth conditions chosen for this study. It appears that the CDP-choline pathway is slightly more efficient in the supply of PC to peroxisomes than the methylation pathway. This result also suggests that different pools of PC may exist in the cell.

In both mutants, the *cki1Δdpl1Δeki1Δ* and the *cho2Δopi3Δ* deletion strains, the PC level of peroxisomes gets close to wild type (see Fig 6). This is not the case for the other organelles tested, because in mitochondria as well as in the endoplasmic reticulum the wild type level of PC was much higher than in mutants, especially in the Δ*cki1Δdpl1Δeki1* strain. It appears that in peroxisomes import and assembly of PC formed by both pathways are more balanced than in the other organelles. Thus, the phospholipid translocation network governing the traffic of phospholipids between synthesizing and accepting organelles may be regulated by individual requirements of the organelles. Not much is known about the transport mechanism of phospholipids to peroxisomes, but it was shown that non-vesicular transport of lipids to peroxisomes may be relevant [38]. Evidence has also been presented that vesicle transport from mitochondria to peroxisomes may exist [39–42]. Membrane contact between different organelles has also been discussed as a prerequisite for lipid translocation (for reviews see refs. [29] and [43–46]), although functionality of contact sites between peroxisomes and mitochondria or endoplasmic reticulum [45] is still a matter of dispute. Such mechanisms, however, may explain the finding that small amounts of CL were found in peroxisomes of all three strains tested in this study. Taking into account the contamination of peroxisomes with mitochondria (see Figs 4 and 5) approximately 30% of CL found in peroxisomes of wild type and *Δeki1Δcki1Δdpl1*; and even 80% of CL found in peroxisomes from the *Δcho2Δopi3* mutant can be regarded as “true” peroxisomal components. This finding is in line with previous observations obtained with isolated yeast subcellular fractions. [35].

**Fig 7:**
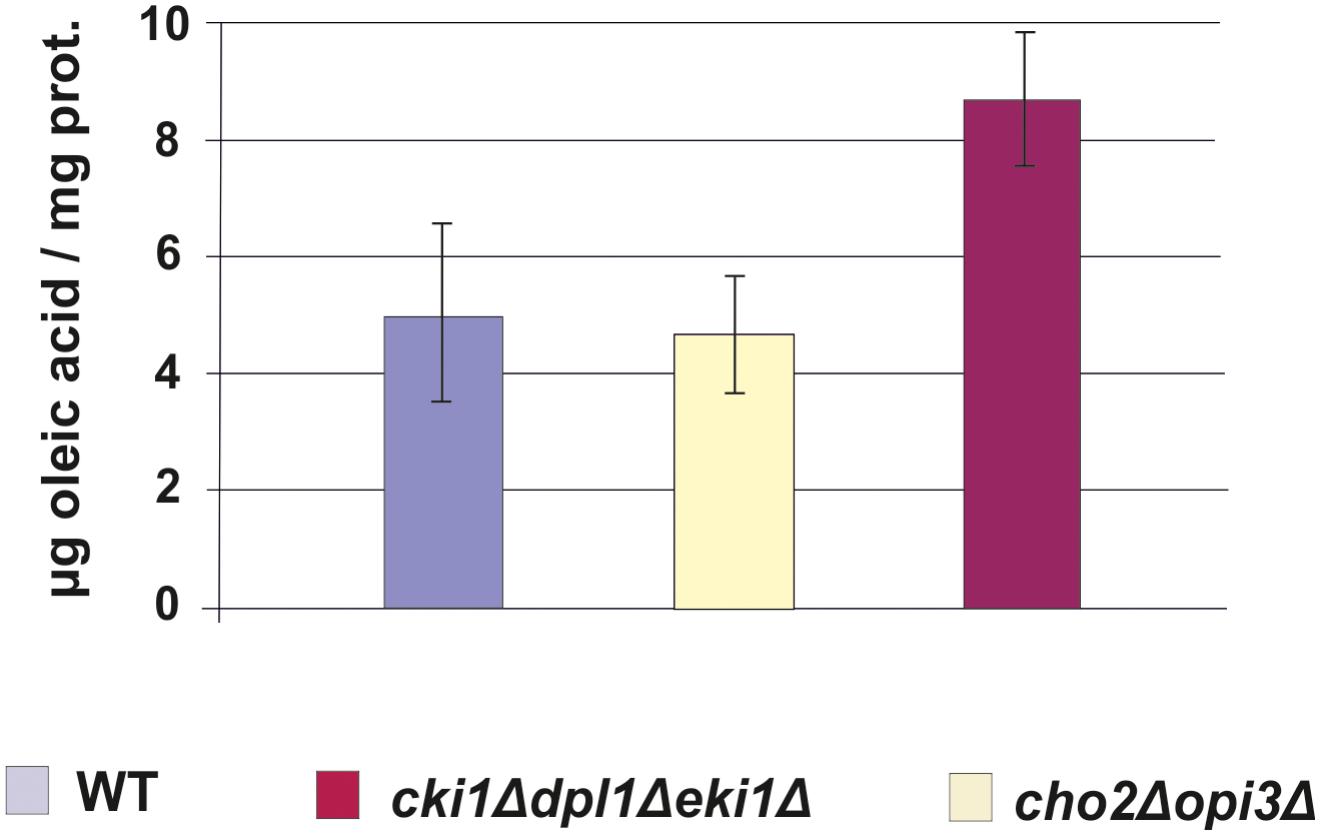
Fatty acids in the homogenate from wild type, *cho2Δopi3Δ* and *cki1Δdpl1Δeki1Δ* yeast strains. Yeast strains used were grown on YPO media, and homogenate samples were prepared after cell disruption. The oleic acid content was quantified as described in the Materials and Methods section.

The impaired growth on oleate media of the two mutants investigated in this study, especially the *cho2Δopi3Δ* strain, may be attributed to changes in the phospholipid composition of organelles. Peroxisomes are of special interest with this respect as they are the organelles which harbor the enzymes of β-oxidation and are required for the utilization of fatty acids as substrates. Thus, the amount of fatty acids present in the strains tested is of considerable importance. As can be seen from Fig 7, however, oleic acid which is the predominant fatty acid in cells grown under the given conditions and the substrate for peroxisomal β-oxidation was not depleted in the two mutant strains compared to wild type. Thus, fatty acid supply to peroxisomes where β-oxidation occurs exclusively in *Saccharomyces cerevisiae* was not limited and energy production not affected.

Although changes in the ratio of saturated to unsaturated fatty acids and the ergosterol to phospholipid ratio have also to be taken into account, the PC to PE ratio seems to have an influence on the anisotropy of peroxisomal membranes. PC and PE differ only in their head groups. However, molecular simulations demonstrated the unique ability of PE to interact strongly with itself and neighboring lipids via inter- and intramolecular hydrogen bonding [46]. This biophysical ability generates a closely packed lipid bilayer with hydrogen tails aligned, decreasing the space occupied by each lipid molecule [47]. The DMPE and PE molecules which are present in a lipid bilayer, form bridges with each other [46–50]. These bridges lead to a higher value of the “main” transition temperature (T_m_) from a fluid to a gel state of the membrane [48]. Furthermore, in model studies insertion of DMPE or PE molecules into a lipid bilayer increased the T_m_ of the mixed PC/PE bilayer systems substantially [49]. Other studies indicated that hydrogen bonds within a cluster remain stable for longer time and that especially PE lipids may diffuse or reorient as groups or clusters rather than individual lipids [50]. As a result, an increased PE to PC ratio leads to a higher transition temperature of a lipid bilayer, i.e. membranes become more rigid. Such effects may apply to organelle membranes from yeast mutants described here.

In summary, our study demonstrates routes of PC transport to peroxisomes in the yeast *S*. *cerevisiae*. Both pathways of PC production, PE methylation and the CDP-choline pathway are able to produce PC for peroxisomes and other organelles. Interestingly, mutations in one of these pathways, each, do not cause extreme defects in peroxisomes indicating that the remaining pathway in a mutant strain can efficiently compensate for the loss of the other biosynthetic route. Despite the flexibility of the yeast, certain consequences of PC depletion such as growth defect or disturbed membrane fluidity became evident pointing out the importance of PC for peroxisomal and other cellular membranes.
