# Correction: Phosphatidylcholine Supply to Peroxisomes of the Yeast *Saccharomyces cerevisiae*

**DOI:** 10.1371/journal.pone.0140080

**Published:** 2015-10-02

**Authors:** Vid V. Flis, Ariane Fankl, Claudia Ramprecht, Günther Zellnig, Erich Leitner, Albin Hermetter, Günther Daum

There is information missing in the Funding section. The correct funding information is as follows: This work was supported by the Austrian Science Fund FWF (projects P21429, P26133 and DK Molecular Enzymology W901-B05 to GD).
